# Engineering Zn^2+^-Based Ternary Heterostructured Photocatalysts via Controlled Reconstruction of Layered Double Hydroxides for Solar-Driven Hydrogen Production

**DOI:** 10.3390/nano16090508

**Published:** 2026-04-23

**Authors:** Denis Cutcovschi, Elena Mihaela Seftel, Gabriela Carja

**Affiliations:** 1 Department of Chemical Engineering, Faculty of Chemical Engineering and Environmental Protection, Technical University “Gheorghe Asachi” of Iasi, Bd. D. Mangeron, 700554 Iasi, Romania; denis.cutcovschi@student.tuiasi.ro; 2 Materials & Chemistry Unit (MATCH), VITO Flemish Institute for Technological Research, Boeretang 200, 2400 Mol, Belgium; elena.seftel@vito.be

**Keywords:** layered double hydroxides structural reconstruction, photocatalytic H_2_ evolution, Zn^2+^-based heterostructure, cascade-type charge transfer mechanism

## Abstract

Active and cost-effective catalysts are essential for obtaining high hydrogen evolution activity. In this paper, we report ZnO/ZnLDH/ZnLDO as a novel ternary Zn^2+^-based heterostructure obtained via the partial structural reconstruction of Zn-based layered double hydroxides (LDH) in a Zn^2+^-containing solution and evaluate its catalytic performance in hydrogen evolution under solar light. The reconstruction strategy induces the formation of a ZnLDH/ZnLDO platform decorated with small ZnO nanoparticles (~3 nm) that are generated in situ during the reconstruction process. The catalysts were extensively characterized for their structure and morphology by XRD, SEM, and TEM/HRTEM, while XPS analysis reveals electronic modulation across the interfaced units. For photocatalytic H_2_ evolution under simulated solar light, the optimized ZnO/ZnLDH/ZnLDO (x = 15%) achieves the highest performance with a hydrogen evolution rate of 1.8 mmol h^−1^ gcat^−1^, outperforming the individual (ZnLDH, ZnLDO) and binary (ZnO/ZnLDH, ZnO/ZnLDO) catalysts. The enhanced activity is attributed to cascade-type charge transfer across the ternary Zn^2+^-based structures, which promotes efficient charge separation. However, excessive reconstruction (high x%) or high-temperature calcination alter the LDH/LDO balance, resulting in decreased photocatalytic activity. This work provides a new idea for designing active multiple interfaced heterostructured photocatalysts by using the partial reconstruction of LDH as a facile and cost-effective approach.

## 1. Introduction

**Scheme 1:**
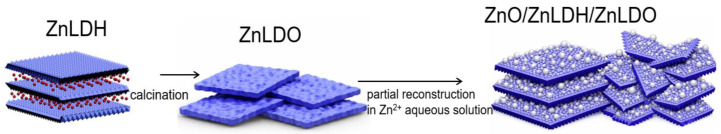
Schematic illustration of the preparation process of ZnO/ZnLDH/ZnLDO heterostructures.

Generation of hydrogen using water is a leading approach to harness abundant solar energy for producing hydrogen as a renewable fuel. However, the practical implementation to increase the photocatalytic hydrogen evolution performance remains severely limited by low solar-to-hydrogen (STH) conversion efficiencies, which typically do not exceed ~1% under realistic operating conditions. Techno-economic assessments indicate that STH efficiencies in the range of 5–9% are necessary to render photocatalytic hydrogen production economically viable [1]. Bridging this substantial performance gap represents a major challenge. Progress toward this target critically depends on the development of more efficient photocatalysts. An advanced catalyst must integrate multiple structural and compositional attributes, including broad and efficient light harvesting, effective charge-carrier generation and transport, and minimized electron–hole recombination to maximize the availability of reactive species [2,3]. Among the strategies reported to suppress charge-carrier recombination in heterogeneous catalysts, surface decoration with metal (Me) or metal oxide (Me_x_O_γ_) nanoparticles has emerged as a promising approach [4,5]. During catalysis, surface-deposited Me/Me_x_O_γ_ nanoparticles play a dual functional role by acting as electron sinks and cocatalytic sites, thereby promoting efficient charge separation [6]. Furthermore, integrating Me/Me_x_O_γ_ nanoparticles into multi-component catalytic platforms enables cooperative interfacial interactions by coupling the functionalities of the assembled components. This generates a broader diversity of catalytically active sites and leads to improved catalytic performance [7]. In the search for versatile catalysts capable of fulfilling these requirements, layered double hydroxides (LDH) have emerged as promising candidates as catalysts or catalyst supports for a broad range of reactions [8,9]. LDH are anionic clays with a two-dimensional layered structure composed of positively charged, brucite-like metal hydroxide sheets with uniformly distributed metal cations, stacked into a lamellar framework and stabilized by interlayer anions and hydrogen-bonded water molecules. Owing to their tunable composition, characteristic porous nanostructure, cost-effectiveness, and scalable synthesis, LDH offer a highly versatile materials platform for a wide range of applications, especially in the field of energy and environment [10]. The hydroxyl-rich surface of LDH might favor the reactants adsorption and promotes both metal-to-metal charge transfer (MMCT) and ligand-to-metal charge transfer (LMCT) processes [11], collectively empowering their potential as efficient photocatalysts. LDH are also well known for their intriguing structural “memory effect”, which enables restoration of the layered structure after calcination-induced collapse and conversion into layered double oxides (LDO) [12]. Although this structural memory effect has been widely exploited as an effective strategy for tuning the properties of reconstructed LDH [13], the deliberate engineering of partial reconstruction to optimize LDH/LDO catalytic platforms remains largely unexplored [14]. In this work, novel ZnO/ZnCuAlLDH/ZnCuAlLDO catalysts were constructed via controlled partial reconstruction of ZnCuAlLDH in an aqueous Zn(CH_3_COO)_2_ solution, as shown schematically in Scheme 1. The resulting ternary catalysts are denoted as ZnO/ZnLDH/ZnLDO. Unlike conventional reconstruction strategies aimed at the complete restoration of the layered structure, the present approach deliberately limits the reconstruction of the LDH framework, leading to the formation of a closely coupled ZnLDH/ZnLDO heterostructure. In addition, small ZnO nanoparticles are generated in situ during the reconstruction process from Zn^2+^ cations present in the reconstruction solution [14] evolving a Zn^2+^-based ternary catalyst with coupled ZnO–ZnLDH/ZnLDO interfaces. The photocatalytic activity was evaluated via H_2_ evolution under simulated solar irradiation as a model reaction.

## 2. Materials and Methods

### 2.1. Catalysts Synthesis

#### 2.1.1. Synthesis of ZnCuAlLDH (Denoted in This Paper as ZnLDH)

ZnLDH was synthesized by a conventional co-precipitation method. Aqueous solutions of Zn(NO_3_)_2_·6H_2_O (35 g), Cu(NO_3_)_2_·6H_2_O (13.7 g), and Al(NO_3_)_3_·9H_2_O (22 g) (Sigma-Aldrich, St. Louis, MO, USA) with a Zn/Cu/Al molar ratio of 2:1:1 were added dropwise to a 1.2 M Na_2_CO_3_ aqueous solution under vigorous stirring at 45 °C. The pH was maintained at approximately 8.5 by the addition of 1.0 M NaOH. The resulting slurry was aged in an autoclave at 45 °C for 24 h, then filtered, thoroughly washed with deionized water, and dried under vacuum at 45 °C [15]. After calcination at 550 °C for 5 h, the resulting mixed oxides were denoted as ZnLDO.

#### 2.1.2. Synthesis of ZnO/ZnCuAlLDH/ZnCuAlLDO Catalysts (Denoted as ZnO/ZnLDH/ZnLDO (x%))

The catalysts were obtained via controlled partial reconstruction of ZnLDH in Zn(CH_3_COO)_2_ (Sigma-Aldrich, St. Louis, MO, USA) aqueous solutions. First, 0.9 g of ZnLDH was calcined at 550 °C for 5 h in air to obtain the corresponding layered double oxides (ZnLDO). The freshly calcined material was rapidly dispersed in 250 mL of an aqueous Zn(CH_3_COO)_2_ solution. The concentration of Zn(CH_3_COO)_2_ was calculated such that the amount of CH_3_COO^−^ in solution corresponded to x% of the anion content theoretically required to reconstruct x% of ZnLDH. In this context, x represents the targeted fraction of ZnLDH reconstructed through the incorporation of acetate anions in the interlayer region. For example, ZnO/ZnLDH/ZnLDO(15) denotes the catalyst obtained using a CH_3_COO^−^ concentration sufficient to reconstruct 15% of the initial ZnLDH. Accordingly, the catalysts were denoted as ZnO/ZnLDH/ZnLDO(x), where x = 15, 25, and 40%. In addition, a reference sample was reconstructed in the presence of CO_3_^2−^ and denoted as ZnLDH/ZnLDO. This sample was prepared following the same protocol, except that reconstruction was carried out in an aqueous Na_2_CO_3_ solution. The reconstruction processes were performed in contact with air at 40 °C for 8 h under vigorous stirring (700 rpm). The resulting solids were recovered by centrifugation and dried under vacuum at room temperature [14].

### 2.2. Characterization Techniques

TEM and HRTEM analyses were performed using JEOL 2100 and JEOL ARM200F NeoARM transmission electron microscope (JEOL Ltd., Tokyo, Japan) operated at an accelerating voltage of 200 kV. A Gatan Orius SC1000 CCD camera (Gatan Inc., Pleasanton, CA, USA) and an Oxford X-Max EDS detector (Oxford Instruments, Abingdon, UK) were used for imaging and elemental analysis. The catalyst morphology, particle size, and composition were evaluated. The samples were prepared by dispersing the powder in ethanol, followed by ultrasonication and deposition onto a carbon-coated copper grid.

XRD analysis recorded using a PANalytical X’Pert PRO MPD diffractometer (PANalytical B.V., Almelo, The Netherlands) with Cu Kα radiation (λ = 0.15406 nm) and a Ni filter, operated at 40 kV and 40 mA. Samples were analyzed as powders and data were collected in the 2θ range of 3–80° with a step size of 0.01° and a counting time of 5 s per step.

X-ray photoelectron spectroscopy (XPS) analyses were performed using a K-Alpha spectrometer (Thermo Fisher Scientific Inc., Waltham, MA, USA) equipped with a monochromatic Al Kα source (hν = 1486.6 eV), operating at a base pressure of 2 × 10^−9^ mbar. Charging effects were compensated using a flood gun, and binding energies were calibrated by setting the C 1 s peak at 284.8 eV as an internal reference. Survey and high-resolution spectra were acquired with pass energies of 200 eV and 20 eV, respectively. Peak deconvolution was performed using a Gaussian–Lorentzian function after background subtraction. The samples were analyzed as powders and were degassed under vacuum prior to measurements.

UV–vis diffuse reflectance (UV–vis DR) spectra were recorded on a Nicolet Evolution 500 UV–vis spectrophotometer (Thermo Fisher Scientific Inc., Waltham, MA, USA) equipped with an integrating sphere for diffuse reflectance measurements of solid samples over the wavelength range of 200–800 nm. The samples were analyzed as powders. The UV–vis diffuse reflectance spectra of the samples were transformed using the Kubelka–Munk function, F(R), to estimate the optical absorption properties. The apparent optical band gap values were determined from Tauc plots by extrapolating the linear region of (F(R)·hν)^2^ versus photon energy (hν), assuming a direct allowed transition, as commonly applied for ZnO-based systems.

The TG–DTG analyses were performed on a TG/DTG/DTA Diamond thermobalance (Thermo Fisher Scientific Inc., Waltham, MA, USA). Approximately 10 mg of sample was placed in a platinum crucible and analyzed under flowing air (40 cm^3^ min^−1^) over the temperature range of [40–800 °C] at a heating rate of [10 °C min^−1^]. The DTG curves were obtained by numerical differentiation of the TG data.

### 2.3. Photocatalytic Experiments

For the photocatalytic experiments, 10 mg of catalyst powder was dispersed in 25 mL of an aqueous methanol solution (H_2_O/CH_3_OH = 80/20, *v*/*v*), where methanol served as a sacrificial agent. After ultrasonic dispersion for 15 min (37 kHz, 150 W), the suspension was transferred into a custom-made 30 mL cylindrical Pyrex reactor equipped with a water-cooling system and purged with argon for at least 20 min to remove dissolved oxygen. During irradiation, the reaction temperature was maintained at 20 °C, and continuous magnetic stirring was applied to prevent photocatalyst sedimentation. Photocatalytic reactions were carried out under full-spectrum irradiation using an Unnasol US 800 solar simulator (Unnasol, Stockholm, Sweden) equipped with a 250 W lamp (UVA: 110 W m^−2^, UVB: 9.5 W m^−2^, visible: 870 W m^−2^; illuminated area with F250 reflector (integrated in the Unnasol system): 220 mm; distance to the illuminated area: 250 mm). The evolved hydrogen was quantified by withdrawing 100 µL gas samples from the reactor headspace and injecting them into a GC-TCD (Agilent 7890A, Agilent Technologies Inc., Santa Clara, CA, USA) operated under isothermal conditions at 50 °C. Hydrogen quantification was performed using a calibration curve constructed with standard H_2_ gas mixtures (1000–10,000 ppm, R^2^ = 0.9993, LOD = 100 ppm). The amount of H_2_ was calculated based on the reactor headspace volume and the ideal gas law. The hydrogen evolution rate was calculated according to:

r_H_2_= n(H_2_)/m*t, where n (H_2_) is the amount of hydrogen produced (mmol), m is the mass of catalyst (g), and t is the irradiation time (h). Gas separation was performed using a molecular sieve capillary column (530 µm internal diameter, 15 m length), and hydrogen was detected using a thermal conductivity detector with argon as the carrier gas, as previously reported [15]. A blank experiment was conducted under similar reaction conditions using the water/methanol solution (80/20, *v*/*v*) in the absence of photocatalyst and under full-spectrum irradiation and no quantifiable hydrogen evolution was detected, under applied conditions. Because full-spectrum solar irradiation was employed, the photocatalytic performance was expressed in terms of the H_2_ evolution rate (mmol g^−1^ h^−1^) rather than wavelength-dependent apparent quantum yield (AQY).

## 3. Results and Discussion

### 3.1. Structural and Morphology Characterization

**Figure 1:**
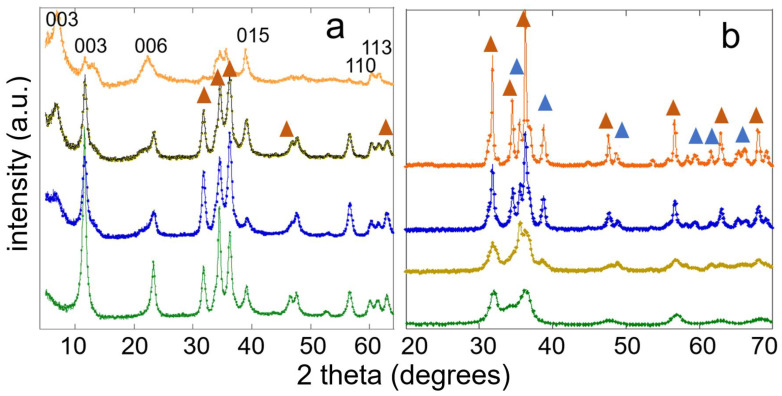
(**a**) XRD patterns of ZnLDH/ZnLDO (♦); Zn/ZnLDH/ZnLDO(15) (♦); Zn/ZnLDH/ZnLDO(25) (♦) and Zn/ZnLDH/ZnLDO(40) (♦). (**b**) XRD patterns of ZnLDH/ZnLDO calcined at 550 °C (♦); Zn/ZnLDH/ZnLDO(15) calcined at 550 °C (♦); ZnLDH/ZnLDO calcined at 750 °C (♦); ZnLDH/ZnLDO(40) calcined at 750 °C (♦); (▲) ZnO; (▲) ZnAl_2_O_4_.

The structural characteristics of the catalysts were investigated by X-ray diffraction (XRD). The as-synthesized ZnLDH (molar ratio: Zn/Cu/Al = 2/1/1) exhibits the characteristic layered structure of LDH [16] with the basal reflection indexed to the (003) plane appears at approximately 11.7° (2θ) with the calculated interlayer spacings (d_003_) of 0.767 nm [17]. Figure 1a presents the XRD patterns of the ZnLDH/ZnLDO and Zn/ZnLDH/ZnLDO(x) catalysts obtained via partial reconstruction in aqueous solutions of Na_2_CO_3_ and Zn(CH_3_COO)_2_, respectively. All samples exhibit diffraction patterns characteristic of hydrotalcite-like LDH materials (JCPDS file No. 38-0486), crystallizing in a hexagonal lattice with rhombohedral R-3m symmetry and displaying well-defined (hkl) reflections [18] revealing the presence of the LDH after the reconstruction. The basal harmonic diffraction peaks observed at low diffraction angles, namely the (003) and (006) reflections, are clearly discernible for all catalysts. However, reflections appearing in the 30–40° (2θ) region reveal also the presence of mixed oxide phases that formed during calcination. This indicates that the reconstruction process is incomplete, leading to the formation of a hybrid LDH/LDO structural platform. Further insight into the partial reconstruction process is provided by analysis of the (003) basal reflection. The sample reconstructed in the presence of CO_3_^2−^ shows a single (003) reflection at 11.7° (2θ), which is characteristic of carbonate-intercalated LDH structures. In contrast, the XRD patterns of Zn/ZnLDH/ZnLDO(x) display two distinct (003) basal reflections, evidencing the coexistence of different interlayer anions intercalated in the reconstructed LDH. The low-angle reflection at ~7.1° (2θ) is consistent with interlayer expansion associated with hydrated acetate-intercalated LDH, as previously reported [19], whereas the reflection at 11.7° (2θ) corresponds to reconstruction mediated by CO_3_^2−^ anions [20]. The competitive intercalation between acetate and carbonate anions during reconstruction is clearly evidenced by the relative intensities of the two (003) basal reflections. As the acetate content (x) increases from 15% to 40%, the intensity of the low-angle (003) reflection at 7.1° (2θ), associated with acetate intercalation [21,22], progressively increases, while the carbonate-related (003) reflection at 11.7° (2θ) [23] correspondingly decreases. This indicates that acetate-intercalated ZnLDH becomes the dominant reconstructed phase with the increase in acetate availability. Although LDH generally exhibit a strong preference for carbonate intercalation due to its high thermodynamic stability [24], in this strategy, the presence of free Zn^2+^ cations in the reconstruction medium might modulate the interionic interactions, thereby favoring acetate intercalation over carbonate incorporation. Overall, XRD analysis indicates that the reconstructed samples exhibit two distinct sets of diffraction features: reflections associated with re-formed ZnLDH, showing interlayer expansions related to different interlayer anions, and reflections marked by orange triangles in Figure 1 assigned to ZnLDO phases originating from calcination. Together, these features confirm that the reconstruction process is only partial, leading to the formation of ZnLDH/ZnLDO heterostructures. Notably, no distinct diffraction peaks attributable to ZnO nanoparticles are detected, most likely due to their small size and high dispersion. To gain further insight into the structure of the resulting LDO phases, the XRD patterns of ZnLDH and ZnO/ZnLDH/ZnLDO(25) catalysts calcined at 550 °C and 750 °C are presented in Figure 1b. As expected, calcination results in the complete loss of the layered LDH structure, since no characteristic LDH reflections are observed. Calcination at 550 °C leads to the appearance of broad diffraction peaks that differ markedly from those of single metal oxides and can be attributed to homogeneously mixed oxide phases containing Cu^2+^, Zn^2+^, and Al^3+^ cations [25]. For ZnO/ZnLDH/ZnLDO(25), a dominant composite mixed-metal oxide phase is formed, accompanied by minor contributions from ZnO and CuO phases. A comparison of the mixed oxide reflections in the 30–40° (2θ) region of Zn/ZnLDH/ZnLDO(25) calcined at 550 °C (Figure 1b) with those observed in the same region for the reconstructed Zn/ZnLDH/ZnLDO(25) (Figure 1a) reveals higher intensities and better-defined diffraction peaks after reconstruction, indicating a higher degree of structural ordering of the LDO phase retained following the reconstruction process.

**Figure 2:**
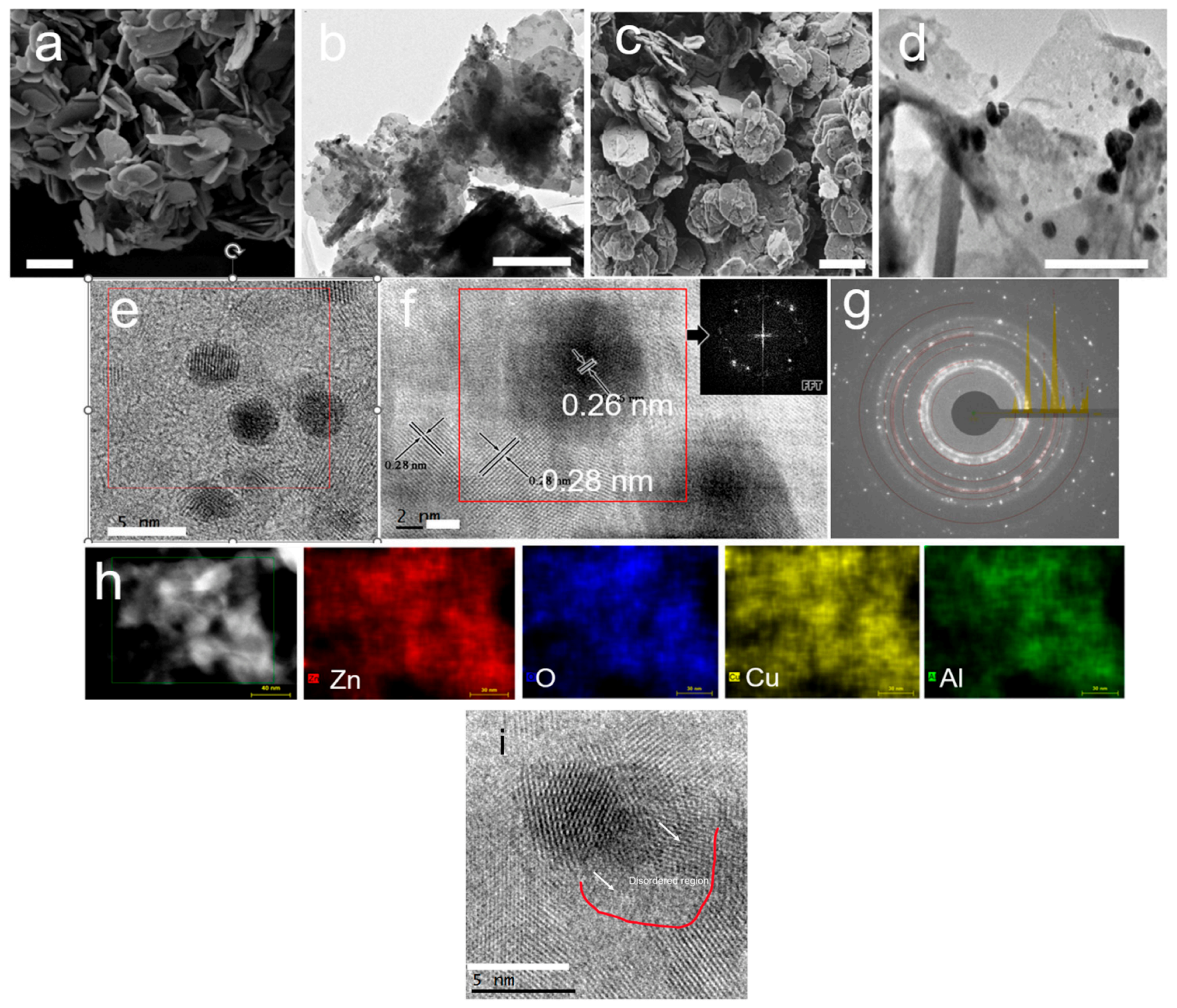
(**a**) SEM image of ZnO/ZnLDH/ZnLDO(15); scale bar is 120 nm. (**b**) TEM image of ZnO/ZnLDH/ZnLDO(15); scale bar is 110 nm. (**c**) SEM image of ZnO/ZnLDH/ZnLDO(40); scale bar is 120 nm. (**d**) TEM image of ZnO/ZnLDH/ZnLDO(25); scale bar is 50 nm. (**e**) High-resolution TEM image ZnO/ZnLDH/ZnLDO(25); scale bar is 5 nm. (**f**) High-resolution TEM image of ZnO/ZnLDH/ZnLDO(15); scale bar is 2 nm. (**g**) (SAED) pattern of ZnO/ZnLDH/ZnLDO(15). (**h**) EDX elemental mapping of ZnO/ZnLDH/ZnLDO(15). (**i**) HRTEM image of ZnO/ZnLDH/ZnLDO(15); scale bar is 5 nm.

After calcination at 750 °C, the XRD patterns of both ZnLDH/ZnLDH and ZnO/ZnLDH/ZnLDO are characterized by well-defined diffraction reflections, which are in good agreement with the standard XRD patterns of well-crystallized hexagonal wurtzite ZnO (JCPDS No. 36–1451), CuO (JCPDS No. 05–0661), and ZnAl_2_O_4_ (JCPDS Card No. 01–1146). In addition, the XRD pattern of ZnO/ZnLDH/ZnLDO exhibits stronger ZnO diffraction peaks [26] than the ZnLDH/ZnLDO sample, indicating the formation of a higher ZnO content after the reconstruction in Zn(CH_3_COO)_2_ solution. The morphology of the reconstructed catalysts was further investigated by scanning electron microscopy (SEM) and transmission electron microscopy (TEM). Representative SEM and TEM images are presented in Figure 2, illustrating the morphological evolution before and after structural partial reconstruction. As shown in the SEM images of ZnO/ZnLDH/ZnLDO(15) and ZnO/ZnLDH/ZnLDO(40) (Figure 2a,c), both samples retain the characteristic LDH morphology [27,28], consisting of closely packed hexagonal platelets with diameters in the range of 70–120 nm. Next, TEM images of ZnO/ZnLDH/ZnLDO(15) and ZnO/ZnLDH/ZnLDO(25) (Figure 2b,d) reveal LDH platelets decorated with numerous small dark points on their surfaces. Higher-magnification TEM images (Figure 2e) reveal that these surface features correspond to spherical nanoparticles with an average diameter of approximately 3 nm, which are densely and uniformly distributed on the LDH platelets. On the other hand, the as-synthesized ZnLDH sample exhibits smooth platelet surfaces, with no nanoparticles observed prior to the reconstruction process. Because of their very small size and high dispersion, these ZnO nanoparticles are not detectable by XRD, but are clearly resolved in the TEM images. Further insight into the ternary catalyst interfaces is provided by high-resolution TEM (HRTEM) imaging of ZnO/ZnLDH/ZnLDO(15) (Figure 2f), which reveals well-defined lattice fringes with interplanar spacings of approximately 0.26 nm and 0.28 nm. The lattice spacing of ~0.26 nm is assigned to the (001) planes of hexagonal ZnO [29], while the spacing of ~0.28 nm corresponds to the (012) planes of the ZnLDH/ZnLDO phases [30,31]. The corresponding selected-area electron diffraction (SAED) patterns (Figure 2g) display concentric diffraction rings originating from overlapping lattice planes, confirming the highly crystalline nature of heterostructure evolved during the partial reconstruction. The presence of bright diffraction spots further indicates the high crystallinity of the individual components. Elemental mapping images (Figure 2h) reveal a dense and uniform distribution of Zn, Al, O, and Cu throughout the selected area, confirming the homogeneous integration of all constituents within the heterostructure.

In addition, the HRTEM image shown in Figure 2i reveals localized regions with distorted and disrupted lattice fringes, indicating the presence of surface structural disorder in the re-formed LDH structure [32]. This provides evidence that the re-formed LDH exhibits a defect-rich surface, demonstrating the ability of the reconstruction process to generate catalytically favorable surface defects in the LDH matrix [33,34]. Hence, SEM, TEM, and HRTEM analyses demonstrate that the partial reconstruction of ZnLDH in a Zn(CH_3_COO)_2_ aqueous solution leads to the formation of a ternary Zn^2+^-based heterostructure, in which ZnO nanoparticles are intimately interfaced with the ZnLDH/ZnLDO matrix, resulting in a defect-rich ZnO/ZnLDH/ZnLDO catalytic platform. The interfaced ternary units are expected to play a key role in promoting efficient charge separation and enhancing the photocatalytic performance of the catalysts.

### 3.2. Surface Characterization by XPS

**Figure 3:**
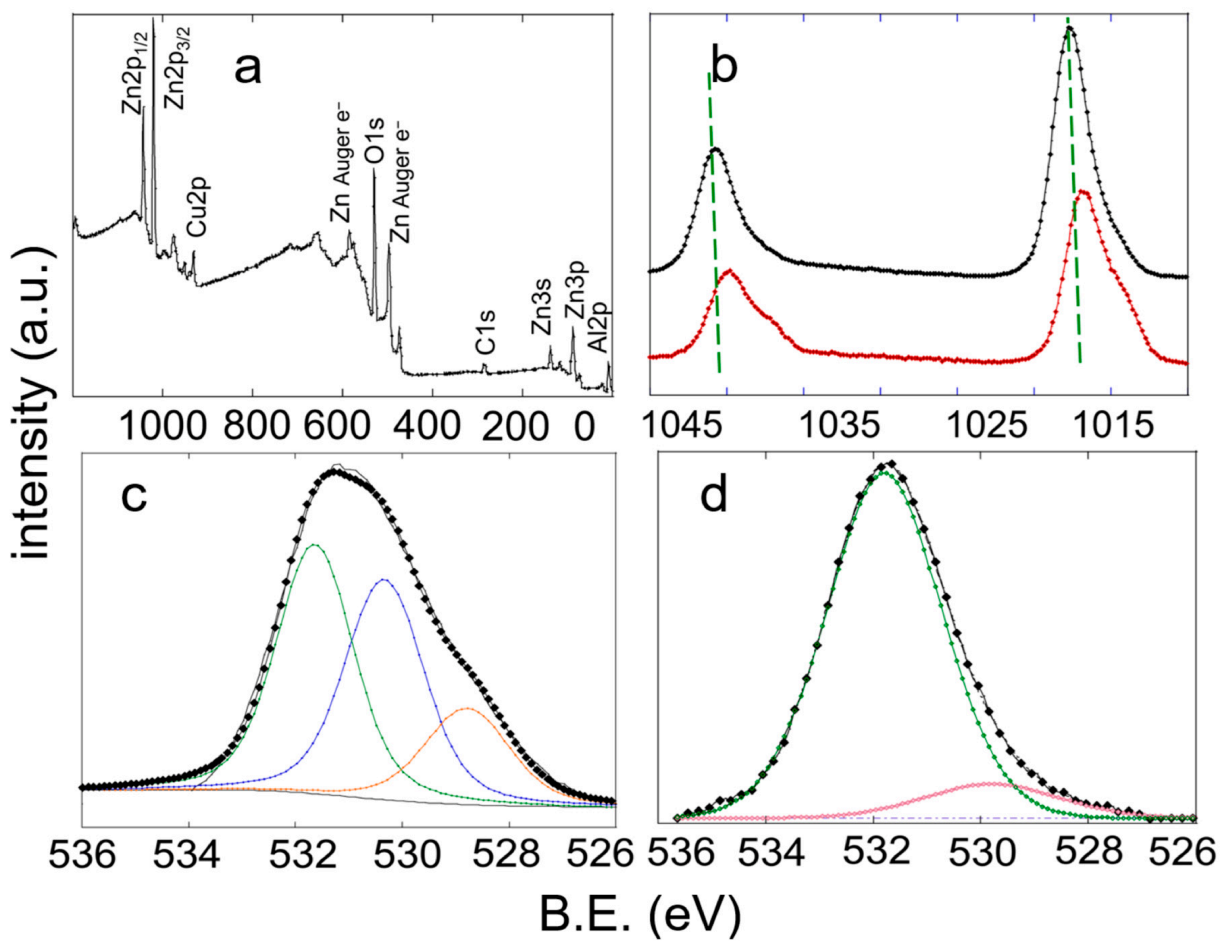
(**a**) XPS survey spectra of ZnO/ZnLDH/ZnLDO(15); (**b**) Zn 2p spectra of ZnLDH (♦) and ZnO/ZnLDH/ZnLDO(15) (♦); (**c**) O 1s spectra of ZnO/ZnLDH/ZnLDO(15), where the blue, green, and red lines correspond to O I, O II, and O III, respectively; and (**d**) O 1s spectra of ZnLDH, where the red and green lines correspond to O I and O II, respectively.

Next, the elemental composition and chemical states of the catalysts were examined by X-ray photoelectron spectroscopy (XPS). As shown in the survey spectra (Figure 3a), the catalyst surfaces contain Zn, Cu, O, and Al, with no additional elements detected within the instrumental detection limits. Figure 3b presents the high-resolution Zn 2p spectra with the characteristic Zn 2p_3_/_2_–Zn 2p_1_/_2_ doublet with a spin–orbit splitting of approximately 23.0–23.2 eV, indicating that Zn is present as Zn^2+^ species. For the parent LDH, the Zn 2p_3_/_2_ and Zn 2p_1_/_2_ peaks are located at 1022.67 eV and 1045.71 eV, respectively. After the partial reconstruction, Zn2p peaks shift to lower binding energies, with Zn 2p_3_/_2_ at 1021.57 eV and Zn 2p_1_/_2_ at 1044.71 eV, while the spin–orbit splitting remains unchanged [35]. This behavior confirms that the oxidation state of Zn is preserved, while indicating a modification of the local electronic environment of the Zn^2+^ species. The observed negative shift in binding energy suggests an increase in electron density at the surface, consistent with the development of interfacial charge redistribution. Such electronic modulation is commonly associated with downward band bending at heterojunction interfaces, which can account for the reduced Zn 2p binding energies [36,37]. Additional information on Zn speciation is obtained from the Zn L_3_M_4_,_5_M_4_,_5_ Auger transition, which is defined by a single, symmetric ZnLMM feature centered at ca. 497 eV on the binding-energy scale, identified in the XPS survey spectra. This feature excludes the presence of metallic Zn and further confirms that Zn remains in the Zn^2+^ oxidation state after reconstruction. Quantitative XPS analysis reveals that the surface concentration of Zn increases progressively from 26 at.% for ZnLDH to 29 at.% for ZnO/ZnLDH/ZnLDO(15), 31 at.% for ZnO/ZnLDH/ZnLDO(25), and 34 at.% for ZnO/ZnLDH/ZnLDO(40). The negative shift of 0.7–1.1 eV observed in the Zn 2p binding energies for the reconstructed catalysts reveals the enrichment of ZnO on the catalyst surface and the appearance of specific Zn–O interfacial environments. Thus, the XPS results complement the TEM observations, providing additional evidence for the successful formation of ZnO/ZnLDH/ZnLDO interfaced heterostructures. Changes in the surface oxygen species are further evidenced by the O 1s spectra (see Figure 3c,d). For the parent ZnLDH, the O 1 s region can be deconvoluted into two main components. The peak at 530.7 eV (OI) is attributed to lattice oxygen bound to metal cations, while the component at 532.5 eV (OII) is usually attributed to mainly hydroxyl groups and the surface-adsorbed oxygen species. In contrast, all ZnO/ZnLDH/ZnLDO catalysts show a broader O 1 s envelope extending toward lower binding energies, which can be resolved into three components. The new low-binding-energy peak at approximately 529.2 eV (OIII) appears, indicating that the reconstructed catalyst surface is enriched in oxide-type Zn–O environments [38]. Hence, the relative contribution of hydroxyl-related oxygen species decreases from 87% for ZnLDH to 54% for ZnO/ZnLDH/ZnLDO(40), indicating partial dehydroxylation while the catalyst surface is enriched in oxide-type species upon reconstruction. The Cu 2p spectra show that the binding energy of the Cu 2p_3_/_2_ core level remains essentially unchanged after the reconstruction, confirming that Cu preserves its Cu^2+^ oxidation state [39]. However, the reconstructed catalysts exhibit a pronounced increase in the intensity of the Cu^2+^ shake-up satellite features. As reported previously [39], this behavior indicates that part of copper oxide species is more present at the catalyst surface after the partial reconstruction, resulting in a more defined oxide-type Cu–O coordination environment of the catalysts and is also evidenced in our previous work [40].

Taken together, the XRD, TEM, and XPS results clearly demonstrate the successful formation of a ternary Zn^2+^-based heterostructure interfacing the closely connected ZnO, ZnLDH, and ZnLDO structures. Such structural integration is expected to facilitate interfacial charge transfer and promote efficient separation of photogenerated charge carriers, thereby contributing to the enhanced photocatalytic performance of the reconstructed catalysts.

### 3.3. Photocatalytic Tests Results

**Figure 4:**
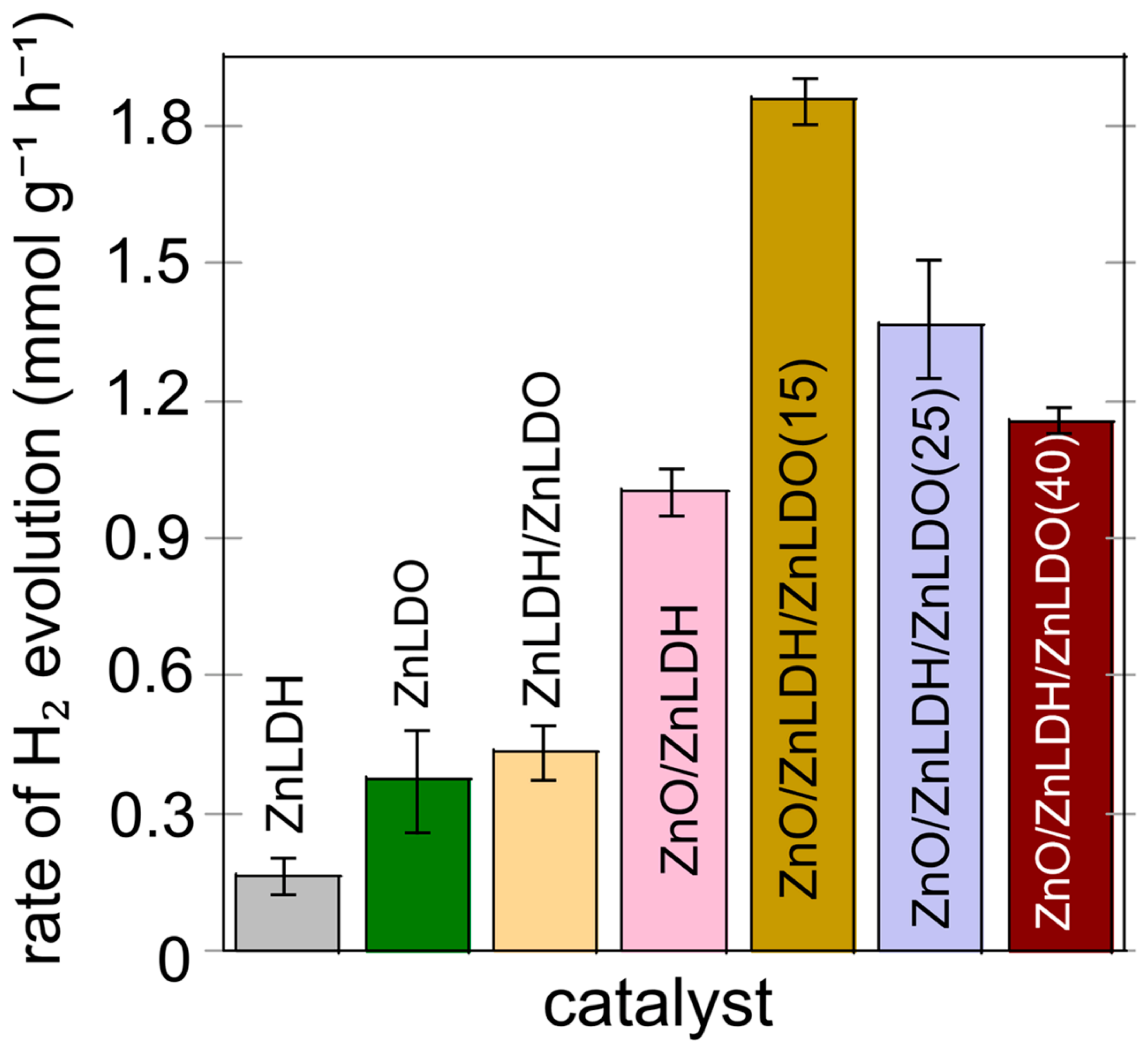
Comparison of the photocatalytic H_2_ evolution rate of different photocatalysts.

Photocatalytic H_2_ evolution was investigated to elucidate the relationship among phase composition, interfacial architecture, and catalytic performance. Figure 4 shows the catalysts activity after 7 h of solar irradiation. It reveals significant differences in activity, indicating the decisive role of heterostructuring the ternary Zn^2+^ phases in governing hydrogen production. The photocatalytic experiments were repeated to ensure reproducibility, and the corresponding error bars, representing the standard deviation, are included. ZnLDH exhibits the lowest activity (0.17 mmol h^−1^ gcat^−1^), indicating limited intrinsic charge separation efficiency and reduction capability. Upon partial transformation into a mixed ZnLDH/ZnLDO platform, the H_2_ evolution rate increases to 0.43 mmol h^−1^ gcat^−1^, demonstrating that the LDH/LDO matrix promotes interfacial charge separation enhancing catalytic activity relative to ZnLDH. To further probe the role of surface Zn^2+^ species, ZnO/ZnLDH(15) was synthesized via impregnation using an aqueous Zn(CH_3_COO)_2_ solution at a Zn^2+^ concentration comparable to that employed for ZnO/ZnLDH/ZnLDO(15). The resulting ZnO/ZnLDH(15) catalyst shows an H_2_ evolution rate of 1.07 mmol h^−1^ gcat^−1^, nearly three times higher than ZnLDH/ZnLDO. This evidences the beneficial role of surface Zn-based species in accelerating reduction kinetics. This suggests that ZnO/ZnLDH interfacial coupling offers a more favorable electron transfer pathway. The highest activity is achieved for the ternary ZnO/ZnLDH/ZnLDO(15), reaching 1.8 mmol h^−1^ gcat^−1^ and reflects the synergistic interplay among the three heterostructured Zn^2+^ units. In the ternary Zn^2+^-based catalyst, the photogenerated electrons originating from ZnLDH domains might be injected into ZnO nanoparticles, which serve as electron accumulation and reduction centers for H_2_ evolution, while ZnLDO domains might act as hole-trapping and oxidation sites, facilitating methanol oxidation and promoting spatial charge separation. Therefore, the catalyst operates as a cascade-type heterojunction enabling the charge migration and suppressing electron–hole recombination.

However, further increasing the Zn^2+^ loading on the surface of ZnLDH/ZnLDO leads to a decline in activity, with the H_2_ evolution rate decreasing to 1.41 mmol h^−1^ gcat^−1^ for ZnO/ZnLDH/ZnLDO(25) and to 1.17 mmol h^−1^ gcat^−1^ for ZnO/ZnLDH/ZnLDO(40). This result indicates that a high density of ZnO nanoparticles may partially shield light absorption by the underlying LDH/LDO platform and block access of reactant molecules to active surface sites. Moreover, surface overloading can reduce the effective availability of oxidation centers within the ZnLDO phase, thereby limiting hole utilization efficiency. At the same time, increasing the concentration of Zn(CH_3_COO)_2_ during the partial reconstruction intensifies the reformation of ZnLDH, progressively shifting the ZnLDH/ZnLDO balance toward a higher ZnLDH fraction. Consequently, the contribution of the LDH phase in the ternary catalyst increases as ZnO/ZnLDH/ZnLDO(15) < ZnO/ZnLDH/ZnLDO(25) < ZnO/ZnLDH/ZnLDO(40), at the expense of ZnLDO domains. Higher amount of ZnLDH might alter the interplay in electron-accumulating and hole-utilizing sites within the ternary structure. Both an optimal ratio between ZnLDH and ZnLDO phases, as well as a controlled amount of surface ZnO nanoparticles, might contribute to increase the catalyst performance.

**Figure 5:**
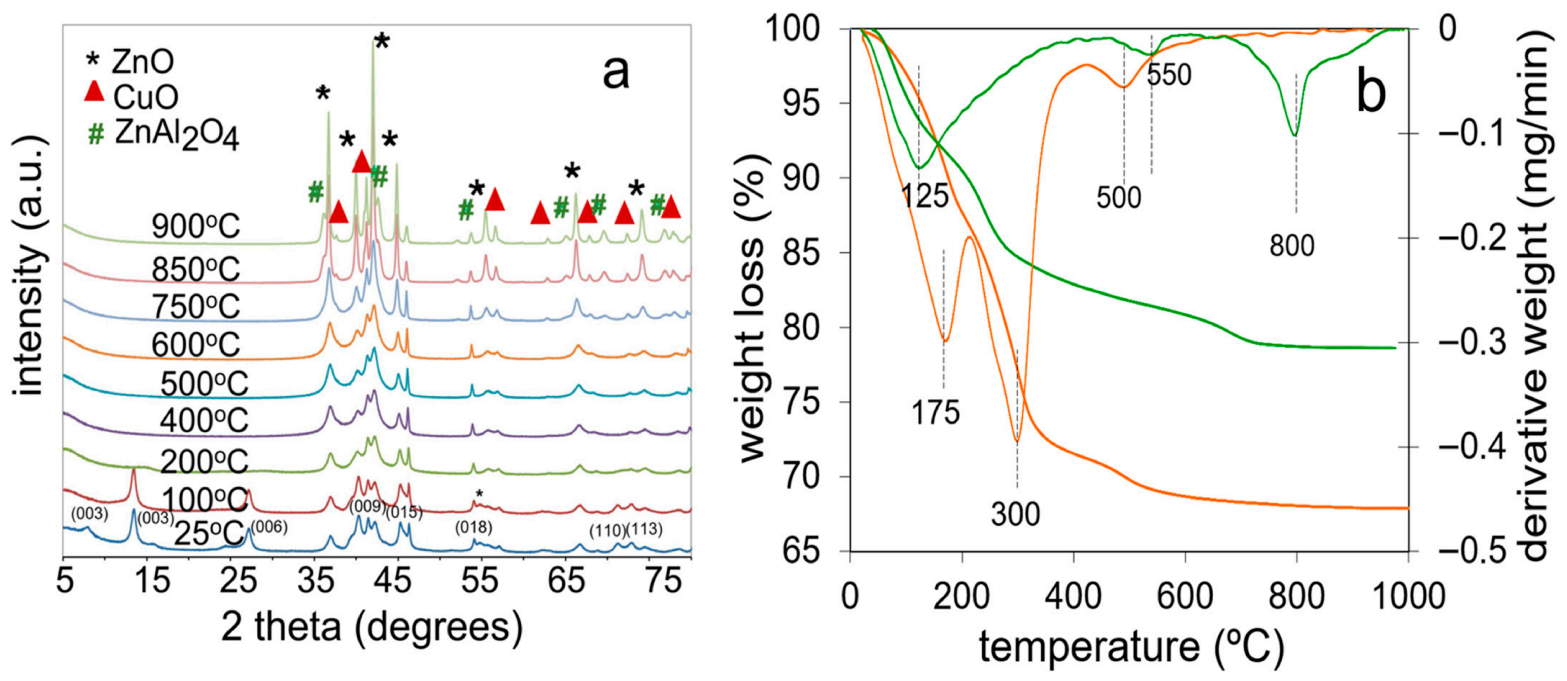
(**a**) In situ XRD patterns of ZnO/ZnLDH/ZnLDO (15) recorded during thermal treatment from 25 to 900 °C. (**b**) The TG-DTA patterns of (♦) ZnLDH and (♦) Zn/ZnLDH/ZnLDO(15).

To further clarify the structure–activity relationship, the effect of progressive LDH collapse was examined using the most active sample, ZnO/ZnLDH/ZnLDO(15). Figure 5a shows the evolution of XRD pattern as a function of temperature revealing the coexistence of ZnLDH and ZnLDO phases in the reconstructed catalyst. After mild treatment at 100 °C, the basal reflections of ZnLDH remain preserved, indicating structural stability under low-temperature conditions. The absence of distinct ZnO reflections confirms that the in situ generated ZnO nanoparticles are highly dispersed and below the XRD detection limit. Upon calcination at elevated temperatures, the reflections of the layered LDH phase progressively disappear, evidencing lamellar collapse. Broad diffraction features characteristic of mixed oxide phases emerge, suggesting poor crystallinity, partial amorphization, and/or high defect density within the oxide matrix. Above 750 °C, these reflections sharpen and intensify, and well-defined diffraction maxima attributable to ZnO (zincite), CuO, and ZnAl_2_O_4_ spinel become distinguishable, indicating the formation of a homogeneous mixed-oxide system. The structural evolution is further supported by the TG–DTG experiments (Figure 5b), which provide complementary insight into the thermal evolution of the ZnO/ZnLDH/ZnLDO catalytic platform. The parent ZnLDH shows three stages of weight loss, typical for LDH-like structures [41]. The first mass loss, from room temperature to 200 °C, is mainly assigned to the removal of interlayer and physisorbed water, accompanied by endothermic effects. The second gradual mass loss, occurring in the temperature range of 200–400 °C, is usually attributed to the dehydroxylation of the brucite-like layers and the decomposition of interlayer anions, leading to the progressive transformation of the LDH structure into mixed oxide phases. At temperatures around 500 °C, the thermal events are related to structural rearrangements and the formation of ZnLDO, consistent with literature reports on Zn-based LDH systems [42]. On the other hand, the ZnO/ZnLDH/ZnLDO catalyst displays a markedly lower overall mass loss and less intense first- and second-stage weight losses. This is in agreement with the partial reconstruction of ZnLDH, which leads to the presence of joined LDH/LDO phases, while similar behavior has been reported for other Me/LDH heterostructures, leading to attenuated dehydration/dehydroxylation events and enriched contribution of the Me oxide phases [43,44]. According to previous findings [42], the slight shift of the dehydroxylation-related transitions toward lower temperatures indicates an increased Zn content in the catalyst, reflecting the lower thermal stability of Zn–OH species compared to Al–OH bonds. Furthermore, a pronounced endothermic peak observed around 800 °C is attributed to structural transformations in non-stoichiometric mixed oxides and phase redistribution involving the ZnAl_2_O_4_ spinel phase detected by XRD [43,45]. Overall, the TG–DTG analysis does not allow a direct quantification of the ZnO content after the partial reconstruction of ZnLDH, as the observed mass losses arise from overlapping processes, including LDH dehydroxylation, decomposition of interlayer species, in situ formation of ZnO nanoparticles, and structural intergrowth within the ZnLDH–ZnLDO matrices.

**Figure 6:**
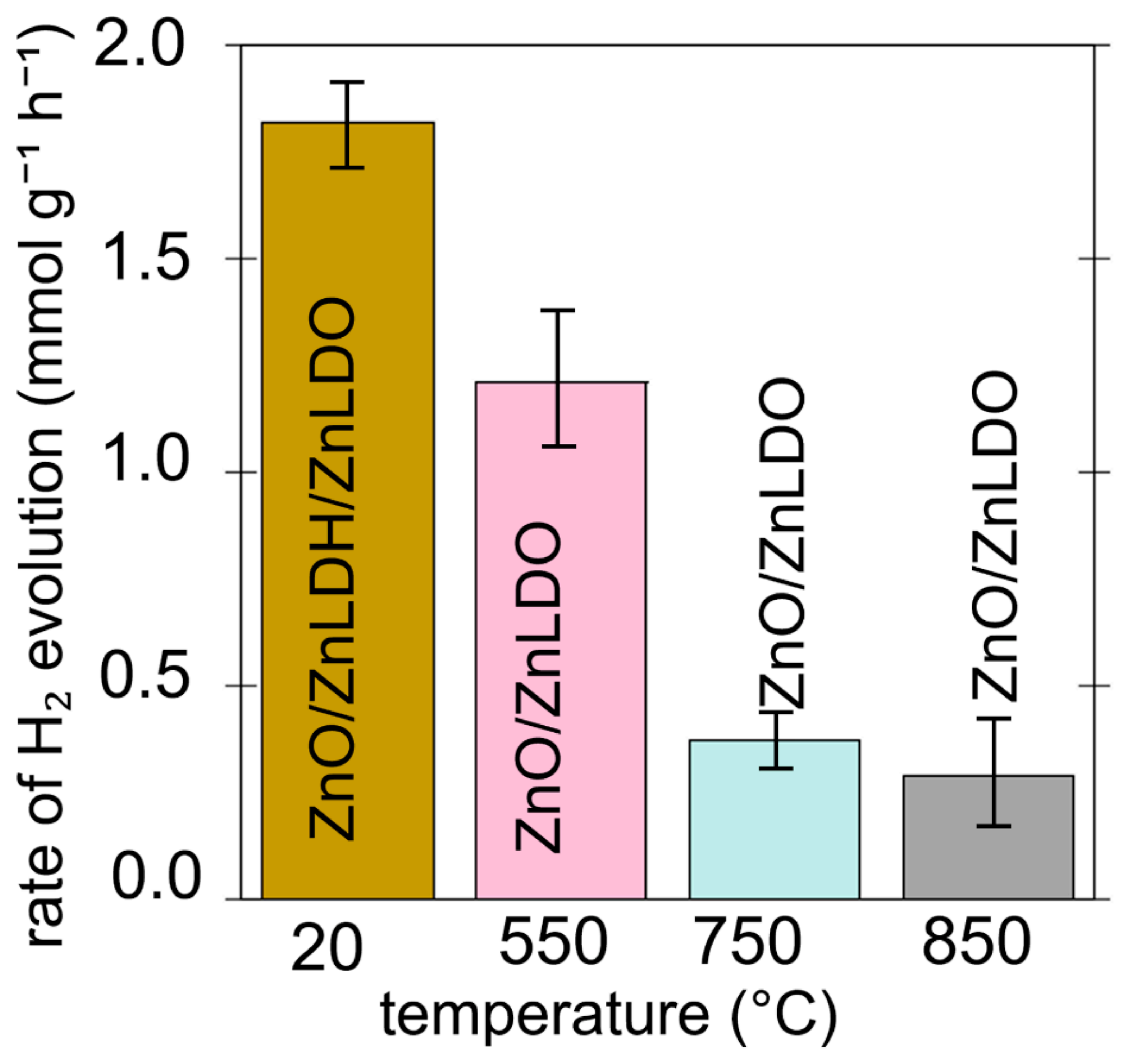
Comparison of the photocatalytic H_2_ evolution rate of ZnO/ZnLDH/ZnLDO, as synthesized and after calcination at different temperatures.

These characteristics are directly reflected in the catalytic performance (see Figure 6). While ZnO/ZnLDH/ZnLDO(15) exhibits the highest H_2_ evolution rate (1.8 mmol h^−1^ gcat^−1^), calcination induces a pronounced decline in activity, with a reduction exceeding 80% at ≥750 °C. Notably, enhanced oxide crystallinity does not translate into improved photocatalytic efficiency. Instead, the cooperative heterointerfaces within the ternary system are crucial for sustaining efficient charge migration. The collapse of ZnLDH domains disrupts this cascade-type architecture, leading to diminished catalytic performance. Overall, the results demonstrate that catalytic efficiency arises from the cooperative interplay among the in situ formed ZnO nanoparticles, the partially recovered ZnLDH, and ZnLDO, where each structural component fulfills a defined role in light absorption, directional charge transport, and redox transformation.

### 3.4. Photocatalytic H_2_ Evolution Mechanism

**Figure 7:**
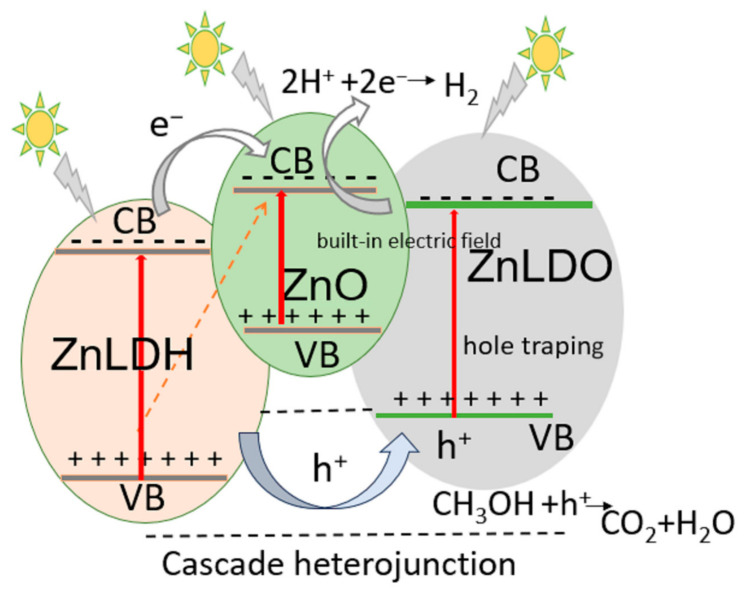
Schematic illustration for the electron transfer and photocatalytic H_2_ evolution mechanism over ZnO/ZnLDH/ZnLDO.

The enhanced photocatalytic performance of ZnO/ZnLDH/ZnLDO(15) arises from a ternary cascade-type heterostructure in which cooperative interfaced ZnO nanoparticles, reconstructed ZnLDH, and residual ZnLDO domains drive efficient charge separation and directional carrier transfer [8,46]. The proposed photocatalytic mechanism is illustrated schematically in Figure 7. Upon solar irradiation, both ZnLDH and ZnO domains can absorb photons and generate electron–hole pairs.

**Figure 8:**
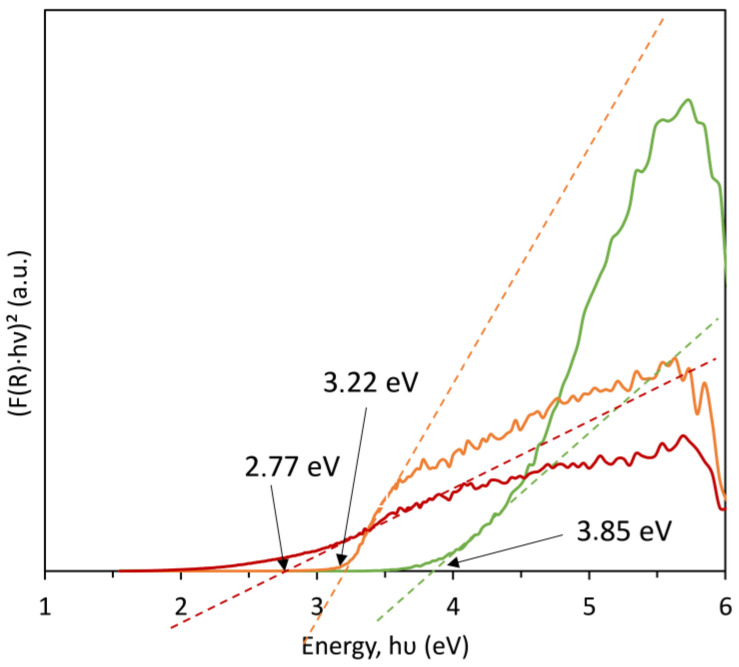
Tauc plots derived from UV–vis diffuse reflectance spectra for (♦) ZnLDH, (♦) ZnLDH/ZnLDO, and (♦) ZnO/ZnLDH/ZnLDO samples.

UV–Vis diffuse reflectance spectroscopy further supports this behavior (see Figure 8). While pristine ZnLDH exhibits a relatively wide apparent optical band gap (3.85 eV) and limited visible-light response, the ZnLDH/ZnLDO sample shows a moderate extension of absorption (3.22 eV), indicating partial modification of the electronic structure. In contrast, the ZnO/ZnLDH/ZnLDO(15) system exhibits the most pronounced absorption broadening and lowest apparent optical transition energy (2.77 eV), demonstrating that the coupling of ZnO with ZnLDH/ZnLDO significantly enhances light harvesting and enables more efficient photoexcitation under solar irradiation. This trend clearly indicates that partial reconstruction and heterostructure formation modify the electronic structure and enhance light absorption, with the ternary ZnO/ZnLDH/ZnLDO(15) system exhibiting the most favorable optical properties for photocatalytic activity. Photoexcitation within the ZnLDH produces conduction-band electrons and valence-band holes. Due to favorable band alignment at the ZnLDH/ZnO interface, photogenerated electrons in the ZnLDH domains are transferred toward the ZnO nanoparticles located at the catalyst surface. ZnO therefore acts as an electron accumulation center, providing active sites for the reduction of protons to H_2_. This directional electron transfer is further supported by XPS analysis, which reveals shifts in the Zn and O binding energies after reconstruction, indicating electronic interaction and charge redistribution between ZnO and ZnLDH phases. The more pronounced binding energy shifts observed in the ternary system compared to the individual and binary samples further indicate stronger interfacial electronic interaction, which is essential for efficient charge separation [46]. At the same time, photogenerated holes migrate toward the ZnLDO mixed-oxide domains that remain after partial reconstruction. These ZnLDO regions possess a defect-rich oxide environment resulting from the calcination process and can effectively act as hole-trapping sites. The presence of these defect-related states is consistent with the extended absorption tail observed in the UV–Vis spectra of the reconstructed samples, suggesting the formation of sub-bandgap states that can participate in charge trapping [31]. The holes are consumed through the oxidation of methanol molecules present in the reaction medium, which suppresses electron–hole recombination. Therefore, the ZnO/ZnLDH/ZnLDO system operates as a cascade-type heterojunction in which electrons and holes are driven toward different domains. Electrons accumulate on the ZnO nanoparticles where hydrogen evolution occurs, while holes migrate to ZnLDO domains where oxidation reactions take place. The ZnLDH phase acts as a light-harvesting and charge-transfer mediator. However, its photocatalytic contribution is significantly enhanced only when coupled with ZnO and ZnLDO, highlighting the importance of the ternary heterostructure. The ZnLDH phase acts as a light-harvesting and charge-transfer mediator between the two components, enabling efficient spatial separation of reduction and oxidation sites. This role is consistent with the improved optical response observed in UV–Vis analysis and the interfacial electronic interaction evidenced by XPS [8,46]. The optimal photocatalytic performance observed for ZnO/ZnLDH/ZnLDO(15) indicates that an appropriate balance between reconstructed ZnLDH, residual ZnLDO domains, and the amount of surface ZnO nanoparticles is essential for maximizing catalytic efficiency. At this composition, sufficient ZnO nanoparticles provide active reduction sites, while the remaining ZnLDO domains effectively trap holes and promote oxidation reactions. When the reconstruction degree increases (ZnO/ZnLDH/ZnLDO(25) and ZnO/ZnLDH/ZnLDO(40)), the relative amount of reconstructed ZnLDH increases, while the proportion of ZnLDO domains decreases. The reduction in ZnLDO regions limits the availability of hole-trapping sites, disturbing the cooperative balance between electron accumulation and hole utilization centers and resulting in lower photocatalytic activity.

Further insight into the importance of the heterostructured architecture is obtained from thermal treatment experiments. Calcination of ZnO/ZnLDH/ZnLDO(15) collapses the layered LDH structure and leads to the formation of crystalline oxide phases such as ZnO, CuO, and ZnAl_2_O_4_.

This structural transformation is consistent with literature reports on LDH-derived mixed oxides, where calcination generates ZnO-based phases and modifies the electronic structure of the material [31]. Although crystallinity increases, the cooperative ZnLDH/ZnLDO heterointerfaces responsible for cascade charge transfer are disrupted as the catalyst transforms into a ZnO/ZnLDO bistructural system. Consequently, charge separation becomes less efficient and the photocatalytic hydrogen evolution activity decreases. Overall, we propose that the cooperative interplay between in situ formed ZnO nanoparticles and the balanced ZnLDH/ZnLDO heterostructure enables a cascade-type charge transfer pathway that promotes efficient carrier separation and enhanced photocatalytic hydrogen evolution.

## 4. Conclusions

This work demonstrates that the controlled partial reconstruction of ZnLDH, driven by the structural memory effect in a Zn^2+^-containing aqueous medium, represents an effective strategy for engineering ternary ZnO/ZnLDH/ZnLDO heterostructured catalysts. The resulting ternary Zn^2+^-based nanoarchitecture enables the formation of cooperative heterointerfaces between in situ generated ZnO nanoparticles and the ZnLDH/ZnLDO matrix, which govern charge separation and transfer.

The enhanced photocatalytic hydrogen evolution is attributed to a cascade-type charge transfer mechanism established across the ZnO, ZnLDH, and ZnLDO components, facilitating directional carrier migration and suppressing recombination. The catalytic performance is governed by the interplay between the LDH/LDO phase ratio and the presence of ZnO nanoparticles, both of which are directly controlled by the degree of reconstruction. This highlights the critical role of intermediate reconstruction states in defining the optimal balance between structural components and interfacial functionality.

These findings establish partial LDH reconstruction as a versatile and scalable materials design strategy for creating multifunctional heterostructured photocatalysts, with broader implications for the development of efficient systems for solar energy conversion and environmental applications.

## Data Availability

Data will be made available upon request.
